# Evaluating semantic control with transcranial magnetic stimulation: a systematic review with meta-analysis

**DOI:** 10.3389/fpsyg.2024.1435338

**Published:** 2024-12-09

**Authors:** Ettore Ambrosini, Silvia Benavides-Varela, Antonino Visalli, Giada Viviani, Maria Montefinese

**Affiliations:** ^1^Department of Neuroscience, University of Padova, Padova, Italy; ^2^Department of General Psychology, University of Padova, Padova, Italy; ^3^Padova Neuroscience Center, University of Padova, Padova, Italy; ^4^Department of Developmental and Social Psychology, University of Padova, Padova, Italy; ^5^IRCCS San Camillo Hospital, Venice, Italy

**Keywords:** controlled semantic cognition, semantic control, semantic representation, semantic aphasia, transcranial magnetic stimulation

## Abstract

**Background:**

This meta-analysis investigates the role of specific brain regions in semantic control processes using Transcranial Magnetic Stimulation (TMS). According to the Controlled Semantic Cognition framework, control processes help manage the contextually appropriate retrieval of semantic information by activating a distributed neural network, including the inferior frontal gyrus, the posterior middle temporal gyrus, and inferior parietal lobule. Lesions in these areas can lead to difficulties in manipulating weakly activated or competing semantic information. Researchers have used TMS to simulate such deficits in healthy individuals.

**Method:**

By synthesizing results from TMS studies that targeted these regions, we aimed to evaluate whether neurostimulation over these areas can effectively impair participants’ performance under high semantic control demands.

**Results:**

Results from different meta-analytical approaches consistently showed no significant effects of TMS, especially after correcting for publication bias. Nevertheless, variability in experimental methodologies was evident.

**Conclusion:**

These findings raise questions about the effectiveness of TMS in simulating deficits in semantic control and highlight the need for methodological improvements in future studies to enhance reliability and interpretability.

## 1 Introduction

Over the course of our lives, we acquire an enormous amount of knowledge about the world, including objects, word meanings, facts, and more, which is not tied to any specific time or place – this is referred to as *semantic representation* (Lambon Ralph et al., 2017; Montefinese, 2019; Tulving, 1972). Information within semantic representation can be available to varying degrees, conveying more salient (dominant) or less salient (non-dominant) aspects of meaning (Montefinese, 2019; Vivas et al., 2020). To highlight context- and task-appropriate aspects of meaning, it is often sufficient to automatically retrieve dominant aspects. However, there are occasions when we must focus attention on non-dominant aspects in a controlled manner or selectively retrieve relevant aspects of meaning while inhibiting irrelevant semantic information (Jefferies, 2013). In these instances, semantic control processes play a crucial role. These processes are distinct from the long-term store of semantic knowledge (Jefferies, 2013; Jefferies and Lambon Ralph, 2006; Noonan et al., 2013) and support our ability to efficiently retrieve and select specific aspects of our semantic representation that are relevant to current goals or context as formulated in the c*ontrolled semantic cognition* (CSC) framework (Lambon Ralph et al., 2017). To borrow an example from Saffran (2000), when thinking about a piano as a musical instrument, keys and pedals (dominant features) are activated automatically. However, in the context of a move, these features become context-irrelevant and must be ignored in favor of features such as weight and size (non-dominant but context-relevant). When the control of semantic information is compromised, individuals lose what Goldstein (1948) called the “abstract attitude” leading to an overreliance on the most immediate and obvious aspects of experience, resulting in deregulated semantic knowledge (i.e., the use of information not pertinent to the context at hand).

This meta-analysis examines over a decade of research using transcranial magnetic stimulation (TMS) to temporarily disrupt control processes in healthy volunteers. It aims to provide causal evidence of the involvement of specific brain regions in these processes, consistent with the CSC framework. In the following sections, this introduction delves into key aspects underpinning our meta-analysis. Section 1.1 provides an in-depth look at the neural mechanisms involved in semantic control, as described by the CSC framework, highlighting the brain regions implicated in control processes. Section 1.2 then explores evidence from neurological patients to illustrate how impairments in semantic control manifest behaviorally and the theoretical perspectives developed to account for these deficits. Section 1.3 introduces the TMS methodology as a tool to investigate semantic control in healthy individuals by creating temporary, controlled disruptions in specific brain regions to simulate patients’ semantic control impairments. Indeed, TMS is a powerful tool that, like lesion and neuropsychological studies, helps researchers understand the causal links between brain regions and their functions. Finally, Section 1.4 outlines the aims and rationale of the current meta-analysis, which is to synthesize findings from TMS studies on key semantic control areas to evaluate the reliability of TMS effects on semantic task performance and assess the implications for the CSC framework.

### 1.1 Neural underpinnings of semantic control processes

According to the CSC framework, semantic cognition activates a distributed neural network (typically left-lateralized), including frontal, temporal, and parietal regions (Binder et al., 2009; Noonan et al., 2013; Jackson, 2021). The distinction between semantic representation and control processes is also reflected in their different brain underpinnings. Semantic representation emerges through learning about the statistical pattern of multimodal experiences with the world. Our knowledge is encoded in modality-specific regions distributed throughout the brain (called ‘spokes’) (Binder et al., 2016; Martin, 2016), while a single transmodal hub, located bilaterally in the anterior temporal lobes (ATL), coordinates the communication among modality-specific ‘spokes’, encodes semantic similarity among items, and stores multimodal semantic representations.

Control processes ensure that task- and context-appropriate information is activated within semantic representation (Jefferies, 2013). The CSC theory posits that both the inferior frontal gyrus (IFG) and the posterior middle temporal gyrus (pMTG) serve to regulate performance in semantic tasks by exerting top-down control over the activation of semantic representations in the ATL (Lambon Ralph et al., 2017). The CSC theory also posits that there would be two types of semantic control processes: (a) *controlled retrieval*, which involves identifying and promoting task-relevant but weak aspects of knowledge; and (b) *semantic selection*, which involves dealing with competition between different aspects of knowledge (e.g., different features of a concept). In controlled retrieval tasks, participants must choose a target based on its relation to a cue (Ambrosini et al., 2023). For strong associations, performance is supported by the automatic spread of activation in the semantic network, while additional control resources are required to recover weak associations, e.g., linking DOG with CAT as animals, compared to DOG with SNAKE (Montefinese et al., 2021). In selection tasks, by contrast, participants must select the target related to the cue while ignoring distractors that are task-irrelevant but strongly related to the cue (Almaghyuli et al., 2012; Montefinese et al., 2020). For example, participants could be asked to select the category (e.g., CUTLERY) to which a cue concept (KNIFE) belongs, while inhibiting a distractor strongly associated with the cue (e.g., SHARP) (Montefinese et al., 2020).

Semantic control processes activate regions in the inferior parietal lobe that partially overlap with the multiple-demand network, which is involved in domain-general executive functions (Duncan, 2010). Noonan et al. (2013) suggested that the dorsal angular gyrus and inferior parietal sulcus (henceforth, inferior parietal lobule, IPL) may contribute to semantic control by directing attention to relevant aspects of knowledge for a given task or context. This is achieved through the adaptive coding of task-critical information (Woolgar et al., 2011), similar to how spatial attention is directed to task-relevant locations. However, the role of these regions in semantic control is debated. Recent evidence has failed to find any involvement of the inferior parietal regions in semantic control specifically (Jackson, 2021).

Nevertheless, as mentioned earlier, pMTG and parts of the left IFG specifically support the control of meaning retrieval (Badre et al., 2005; Davey et al., 2016). While the ventral parts of IFG and pMTG seem to be involved in the controlled retrieval of weak information only, the posterior part of IFG appears to be involved when the demands for semantic selection are high (Badre et al., 2005).

### 1.2 Deficits in semantic control processes in neurological patients

The study of semantic control originated from evidence of deficits observed in neurological patients. Indeed, following the seminal work of Warrington and Shallice (1979), a long tradition of neuropsychological studies on post-stroke patients investigated the deficit in accessing and recovering semantic information (Campanella et al., 2013; Warrington and Mccarthy, 1983). Since then, four main theoretical perspectives have been proposed to explain the behavioral phenomena associated with deficits in semantic access. However, although all of these theories share the theme that, in patients with post-stroke aphasia, semantic representation is intact but the retrieval of information from this representation is impaired (but see also Rapp and Caramazza, 1993), no single existing perspective can account for all of their behavioral phenomena (for a review on the different alternative accounts of behavioral deficits in post-stroke aphasia, see Mirman and Britt, 2014). In this meta-analysis, we will investigate the roles of specific brain regions implicated in semantic control. We will do so within the CSC framework, which takes into account both the concepts of representation and control and integrates them under the label of semantic cognition. Semantic representation and control can be impaired separately, yielding dissociations between semantic dementia (characterized by degradation of the conceptual representation following anterior temporal lobe atrophy) and semantic aphasia (SA), which is highly relevant for the present work, that results in deficits in semantic control and difficulties in manipulating semantic knowledge in the context of an intact semantic representation (Corbett et al., 2009a; Corbett et al., 2009b; Jefferies and Lambon Ralph, 2006; Rogers et al., 2015). SA patients show inconsistent performance in different semantic tasks that tap the same concepts (Campanella et al., 2013; Jefferies and Lambon Ralph, 2006) and have difficulties in inhibiting dominant distractors or retrieving distant relationships between concepts and less relevant meaning dimensions (Noonan et al., 2013). When asked to name pictures, SA patients show improvement following cues that provide external constraints on retrieval (Corbett et al., 2011; Jefferies et al., 2008b) and exhibit equivalent impairment across modalities when control demands are kept constant (Corbett et al., 2009a; Corbett et al., 2009b; Gardner et al., 2012), indicating that their disorder does not stem from a loss of knowledge, but rather depends on control demands. SA patients perform worse when pictures are presented in related stimulus sets than in unrelated stimulus sets in blocked cyclic paradigms, and this difference increases as the number of stimulus repetitions increases (i.e., a negative serial position effect) (Gardner et al., 2012; McCarthy and Kartsounis, 2000). This results in generally inconsistent performance over repetitions of the same items across several cognitive tasks, highlighting a semantic access disorder rather than an impairment of semantic representation.

Patients with SA are better at retrieving the meaning of highly imageable items (Jefferies et al., 2008a), and they do not show a benefit from concept frequency (Jefferies et al., 2008b). Rather, they often exhibit absent or reverse frequency effects (Almaghyuli et al., 2012; Hoffman et al., 2011): high-frequency words exert greater demands on cognitive control probably because they tend to appear in a broader range of linguistic contexts and have more variable meanings. Finally, the non-semantic executive control deficits in SA patients parallel the problems in the semantic domain (Jefferies and Lambon Ralph, 2006).

### 1.3 Fundamentals of TMS methodology

The ability of healthy individuals to control semantic retrieval and selection can be disrupted using inhibitory TMS protocols, that is, offline repetitive low-frequency TMS, continuous theta burst TMS, or online multiple-pulse TMS (Beynel et al., 2019). These protocols can induce a so-called virtual lesion in neurologically intact participants. TMS produces focal effects, enabling comparisons of the roles of different brain regions within the same individuals and distinguishing between brain regions that are often damaged together in patients.

When applied over a specific cortical region, a train of high-intensity magnetic pulses can temporarily impair normal functioning of that region. By observing the effects of these changes on behavior or cognitive functions, researchers can infer the causal role of those brain areas. In this respect, TMS technique enables comparisons between the performance of healthy participants under TMS and patients with lesions in areas involved in semantic control. TMS can be administered using different paradigms that align with the two main protocol categories: offline and online stimulation (Beynel et al., 2019). In offline protocols, task performance is evaluated before and after TMS administration. In online protocols, TMS stimulation is applied at specific time points while participants are engaged in a cognitive task, and the immediate effect on their performance is assessed. Furthermore, TMS experimental designs employ two basic types of control conditions. To test the neuroanatomical specificity of a region, available methods include: (i) stimulating a site unrelated to the function being studied, (ii) using a sham stimulation condition that mimics TMS nonspecific effects without inducing any neural modulation, and (iii) using a no-stimulation condition, which represents a weaker control as it does not account for the sensory confounds of TMS conditions. To assess the function of a specific region, (iv) the control task (or condition) method is more effective. This involves comparing the effects of TMS on experimental and control tasks, with the prediction that TMS should affect the target task involving the cognitive process of interest but not the control task (Jahanshahi and Rothwell, 2000).

### 1.4 The present study

To simulate the deficits observed in SA patients, several studies have applied TMS on healthy volunteers to temporarily inhibit activity in specific brain regions, including the IFG, pMTG, and IPL (Davey et al., 2015; Hoffman and Crutch, 2016;Hallam et al., 2016; Häuser et al., 2016; Krieger-Redwood and Jefferies, 2014; Medaglia et al., 2018, 2021; Teige et al., 2018; Whitney et al., 2011, 2012; Zhang et al., 2019). These TMS interventions were designed to assess their impact on semantic control task performance in a controlled experimental context. However, despite the increasing number of TMS studies, a comprehensive systematic review is still lacking. TMS effects tend to be subtle, studies are often underpowered, and findings may not consistently replicate across different laboratories. Thus, the question remains: Do inhibitory TMS protocols reliably induce significant performance decline in demanding semantic decisions among healthy volunteers, consistent with CSC predictions? To address this question, we conducted a meta-analysis of all existing TMS studies targeting the IFG, pMTG or IPL. We did this within the CSC framework, which takes into account both the concepts of representation and control and integrates them under the label of semantic cognition.

## 2 Method

This meta-analysis was not registered, and no protocol was prepared. However, it adhered to the Preferred Reporting Items for Systematic Reviews and Meta-Analyses (PRISMA) guidelines (Page et al., 2021a, 2021b). Various strategies were used to find relevant articles and then several criteria were applied to determine whether a study could be included in the meta-analysis.

### 2.1 Search for the literature

A computer-based search was performed using the electronic bibliographic databases PubMed, Scopus, Web of Science, and PsycInfo for articles containing the following terms in their title, abstract, or keywords: (“*semantic cognition” OR “semantic control” OR “semantic selection” OR “controlled retrieval”*) *AND* (*TMS OR “transcranial magnetic stimulation” OR TBS OR “theta burst stimulation”*). It should be noted that literature search on PubMed was limited to titles and abstracts, as keywords cannot be included in the search. The search was limited to peer-reviewed articles published up to August 2024. Further candidate studies were identified by checking the reference lists of reports that passed the screening process and those of previous reviews and meta-analyses on semantic control processes (Hoffman and Morcom, 2018; Jackson, 2021; Mirman and Britt, 2014; Lambon Ralph et al., 2017; Noonan et al., 2013).

### 2.2 Eligibility criteria

Different eligibility criteria were used according to the prespecified hierarchy detailed in what follows.

1. Only primary studies reporting original results were included (e.g., no reviews or meta-analyses). Moreover, only studies collecting and analyzing quantitative data that were published in peer-reviewed journals and were available in English were considered. Other eligibility criteria were assessed using the PICO framework (Patient, Intervention, Comparison, Outcome) (Schardt et al., 2007), as follows.
2. (Population): we included studies on healthy adult participants (18 years of age or older);
3. (Intervention): we included studies using inhibitory TMS to cause a virtual lesion (see above), with the TMS targeting the IFG and/or pMTG and/or IPL;
4. (Comparison): we considered studies employing an experimental design that included at least a dual contrast (i.e., at least a 2-factor statistical design) to control for (i) the specific effect of TMS stimulation (ii) on semantic control ability. In other words, we considered studies (i) contrasting a condition with the inhibitory TMS with at least one TMS-related control condition (no TMS, or sham simulation, and/or TMS stimulation over a control site) (ii) on semantic control processes (i.e., contrasting a condition with high semantic control requirements with a condition with low semantic control requirements and/or a non-semantic control task). To determine the conditions with high semantic control requirements, we adopted the same contrasts employed in Jackson’s (2021) meta-analysis. Across all these contrasts, the level of semantic control required varied in several ways: (a) Some tasks emphasized subordinate or less frequent aspects of meaning (e.g., weaker associations, subordinate homonyms). (b) Other tasks demanded inhibition of prepotent responses or increased interference from competitors (e.g., more distractors or greater similarity to distractors). (c) Certain tasks focused on resolving incongruent meanings or ambiguity (e.g., semantic violations, homonym ambiguity). (d) Some tasks intentionally reduced contextual support for determining meaning (e.g., context surprisal, unpredictability). (e) Finally, specific tasks required flexible switching between different meanings or contexts (e.g., alternative uses of task, or switching instructions);
5. (Outcome): we considered the studies testing the specific TMS-induced increase of the semantic control-related effects (i.e., a performance worsening when semantic control requirements were higher) on participants’ response times, which are a more sensitive measure of TMS-induced detrimental effects on participants’ cognitive performance (which are assumed to be caused by a disturbance in the normal functioning of the stimulated region, rather than its inactivation; Pascual-Leone et al., 2000).

### 2.3 Study selection

**Figure 1:**
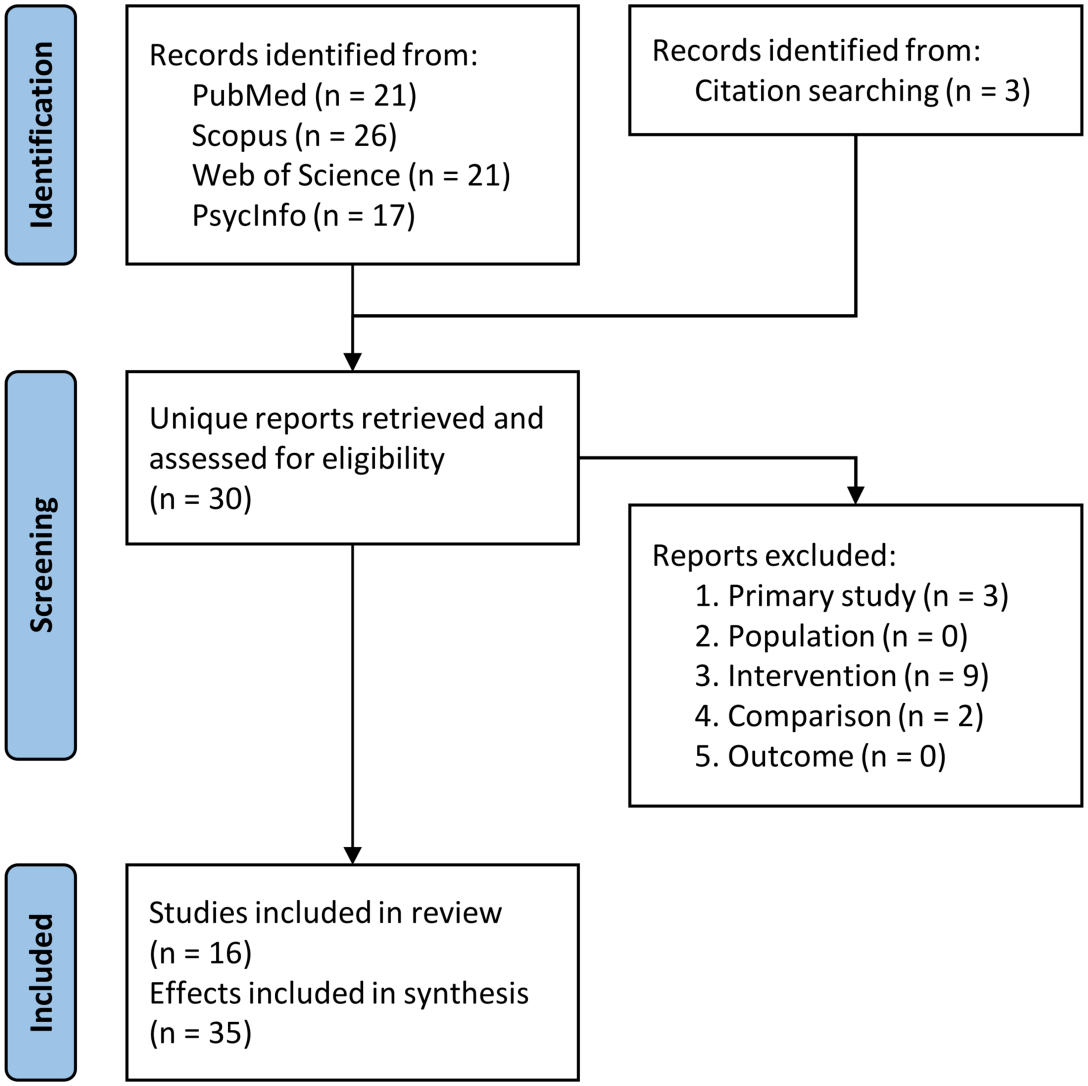
Flow chart of the selection process for the included studies. See the main text for the description of the exclusion criteria.

In our meta-analysis, one author (EA) performed the electronic database searches and the first round of screening to exclude duplicate records. Subsequently, the reports (full-text articles) for the resulting unique records (see Figure 1) and those identified via citation searching were retrieved and two authors (EA and MM) independently assessed them to determine their eligibility. In cases of disagreement between the two reviewers, a third reviewer (SBV) was there to solve them. Notably, no discrepancies arose between the initial screeners (EA and MM).

### 2.4 Data extraction

The effectiveness of TMS over the IFG, pMTG, or IPL in disrupting semantic control processes was investigated by comparing participants’ semantic control ability across different conditions. Specifically, the experimental effects were measured as the difference in response times between a high semantic control condition and at least one control condition/task with no or low semantic control demands (as detailed above). These differences were then compared between the active TMS condition and a control condition (e.g., no TMS, sham TMS, or TMS applied to a control site). The resulting outcome thus reflects the interaction between the specific TMS effects and the varying levels of semantic control requirements, with positive values indicating that TMS decreased semantic control (i.e., increased the performance cost in high semantic control conditions).

For the statistical assessment of the participants’ specific semantic control-related effects, we considered the effects tested by employing either (1) a 2-level SCP (semantic control process) factor, contrasting conditions with higher vs. lower semantic control requirements within the same experimental task; (2) a 2-level TASK factor, contrasting a task with semantic control requirements with a non-semantic task; (3) both SCP and TASK factors (i.e., an SCP × TASK interaction). To ensure using the best estimation of the specific semantic control-related effects, whenever possible we preferred to extract the effects derived from the SCP × TASK interaction, assuring a better control of unspecific performance effects, followed by the SCP factor, providing a more direct effect over the TASK factor, which in turn provides the least control of the specific semantic control-related effects. Moreover, for the statistical assessment of the specific TMS-related effects, we considered the effects tested by employing either (1) a 2-level TMS factor, contrasting the active TMS condition with either a no-TMS or a sham stimulation condition; (2) a 2-level SITE factor, contrasting the active TMS condition over one of the brain regions of interest (i.e., IFG, pMTG, and IPL) and the same TMS stimulation over a control site (e.g., the vertex); (3) both TMS and SITE factors (i.e., a TMS × SITE interaction). To ensure using the best estimation of the specific TMS-related effects, whenever possible we preferred to extract the effects derived from the TMS × SITE interaction, assuring a better control of unspecific TMS effects, followed by the SITE factor, providing a more controlled effect over the TMS factor, which in turn provides the least control of the TMS-specific effects. Therefore, the effects of interest derived from at least a 2 × 2 interaction between a TMS/SITE factor and an SCP/TASK factor, and at best a 2 × 2 × 2 × 2 TMS × SITE × SCP × TASK interaction. When the eligible studies employed a different statistical design (e.g., using a 3 × 2 design to contrast a semantic control-related effect–derived from conditions with higher vs. lower semantic control requirements–across three TMS conditions–active TMS vs. no TMS vs. TMS over a control site) we followed the prespecified hierarchy we just described to extract the 2 × 2 SITE × SCP effect.

When multiple experimental effects of interest were reported (e.g., when TMS was administered at multiple active sites, or when more semantic control tasks were performed), all of them were extracted and included in our meta-analytic models.

For each included effect, two authors (MM and EA) independently extracted the relevant outcome data for the statistical comparison reflecting the experimental effects of interest. In doing this, we again followed a prespecified hierarchy: When available, the F statistics (or the T statistics) and related degrees of freedom were extracted (and used to compute the corresponding exact *p*- and *z*-values) for the statistical comparisons described above. When these statistics were not available, the means (M) and standard deviations (SD) were extracted for the outcome of interest. Specifically, we extracted the mean (and related SD) of the difference between high vs. low/no semantic-control scores for both the active TMS group/condition and the control/sham groups/conditions (M1 and SD1 and M2 and SD2, respectively) as a measure of the semantic control performance. If SD were unavailable, standard errors (SE) were extracted. When these data were presented only as graphs, *WebPlotDigitizer*^1^ was used to extract M and SD/SE estimates from the available graphs.

Based on these outcome data, we computed the corresponding effect sizes (Hedge’s *g*, a standardized mean difference which is equivalent to the bias-corrected version of Cohen’s *d*) for the effects of interest, as well as the corresponding sampling variance (V), SE and 95% confidence interval (CI_95%_). Positive *g* values indicated a TMS-dependent increase of the performance cost in the conditions with higher vs. lower semantic control requirements, that is, a TMS-dependent impairment in semantic control ability. For within-participants designs, computing the *g* (and *d*) requires taking into account the correlation (*r*) between the two repeated-measure semantic control-related effects (M1 and M2), because the pooled SD is computed as the square root of (SD1^2^ + SD2^2^–2 × *r* × SD1 × SD2). However, this *r* value was never reported in the included within-participants studies, so we conservatively chose to use a value of *r* = 0.5. However, we also performed a sensitivity analysis by replicating all the analyses using the values *r* = 0, 0.25, and 0.75. The effect size and variance calculation were performed using R and the functions *escalc* and *vcalc* from the *metafor* package.

For each included effect, two authors (MM and EA) independently extracted the information about the corresponding report, the sample size used in the statistical analyses, the study design, the type of task or TMS control contrast, the TMS stimulation parameters, and the analyses and outcomes. Any discrepancies were solved by discussion.

### 2.5 Data analysis

#### 2.5.1 Risk of bias assessment

Following the Cochrane guidelines (Higgins et al., 2011), the methodological quality of the studies was assessed using the RoB-2 tool (Sterne et al., 2019). The tool is structured into six domains through which bias could be introduced into the outcome. These were identified based on empirical evidence and theoretical considerations. Because the domains cover all types of bias that may affect experimental results, each domain is mandatory, and no additional domains should be added. The six domains are: (1) bias arising from the randomization process; (2) bias due to period or carryover effects; (3) bias due to deviations from intended interventions; (4) bias due to missing outcome data; (5) bias in the measurement of the outcome; and (6) bias in the selection of the reported result. For instance, the following signaling questions are used to determine the risk of bias for each domain: (1) “Was the allocation sequence concealed until participants were enrolled and assigned to interventions?”; (2) Was there sufficient time for any carryover effects to have disappeared before outcome assessment in the second period? (3)“Were participants aware of their assigned intervention during the trial?”; (4) “Were data for this outcome available for all, or nearly all, participants randomized?”; (5) “Was the method of measuring the outcome inappropriate?”; and (6) “Were the data that produced this result analyzed in accordance with a prespecified analysis plan that was finalized before unblinded outcome data were available for analysis?

For each category of risk, two investigators (MM and EA) independently answered multiple questions for each domain with a 5-level multiple choice answer (yes, probably yes, no, probably no, and no information). Any discrepancies were solved by discussion. The RoB 2 tool included an algorithm for automatic calculation of the domain-specific level of bias and for overall bias. A study was characterized with a low risk of bias when all domains were considered to have a low risk of bias; with some concerns when at least one domain took a “some concerns” evaluation; with a high risk of bias when at least one domain was considered to have a high risk of bias or when at least three domains took a “some concerns” evaluation. It is important here to note that we did not include the domain of the randomization process in assessing the overall risk of bias, because the use of TMS makes it practically impossible to prevent investigators and participants from knowing the allocated intervention (e.g., experimental vs. sham or no stimulation); therefore, most of the included studies (13 out of 16) would have been rated with a high risk of bias due to this issue.

#### 2.5.2 Risk of publication bias

There are several methods to assess the presence of publication bias. Publication bias was first examined with a funnel-plot-based method for the effect sizes, and the eventual presence of this bias was then corrected by using the trim-and-fill method (Duval and Tweedie, 2000). To test for potential small study bias, we also examined the presence of funnel plot asymmetry using the rank test and the Egger’s regression test (Egger et al., 1997). In the funnel plot, more precise estimates are located at the top near the combined effect size, whereas less precise estimates are located at the base of the funnel plot. If there is no publication bias, the studies would be expected to be symmetrically distributed on both sides of the combined effect size line. In case of publication bias, the funnel plot may be asymmetric since the absence of studies would distort its distribution on the graph. The trim-and-fill method examines this asymmetry and, with a rank-based data augmentation procedure, estimates the number and location of missing studies, adjusting for the possible effects of missing studies. If the conclusion of the meta-analysis remains unchanged after adjustment for publication bias, the results can be considered reasonably robust, excluding publication bias.

However, the trim-and-fill method only corrects for publication bias based on observed effect size and not based on whether an effect was significant (Simonsohn et al., 2014), and it does not yield corrected meta-analytic effect size estimates that are close to the true effect size when publication bias is based on the *p*-value of the study (Peters et al., 2007; Terrin et al., 2003). Therefore, we further examined publication bias using selection models based on the *p*-values of the included studies (Hedges, 1992; Iyengar and Greenhouse, 1988; Vevea and Hedges, 1995). These selection models use weighted distributions to estimate the probability that non-significant studies were included in the meta-analysis (the publication bias) based on the average effect estimate. If non-significant results are less likely to be published than significant ones, this approach produces an adjusted average effect estimate that accounts for the estimated publication bias by giving more weight to the studies included in the intervals with lower publication probability (which are usually the non-significant ones). Selection models also have the advantage of working well even under high heterogeneity (Carter et al., 2019) and are based on a well-founded model of the publication process and how publication bias actually occurs (i.e., research studies are selected for publication based on the observed statistical significance; Ferguson and Heene, 2012; Masicampo and Lalande, 2012). We initially specified a two-sided selection using p-value cutoffs driving publication bias for significant and marginally significant studies as *p* = 0.05 and 0.1. We also used the selection model to test for publication bias by comparing the unadjusted and selection model using a likelihood ratio test. We used both frequentist selection models and a robust Bayesian meta-analysis (RoBMA, Maier et al., 2023) that combine selection models to model averaging (for details on Bayesian model averaging, see Gronau et al., 2017).

Finally, publication bias was examined using the *z*-curve analysis (Bartoš and Maier, 2020) on the z scores computed from the extracted *p*-values of the included effects, using the *zcurve* R package (Bartoš and Maier, 2020; Bartoš and Schimmack, 2020; Schimmack and Brunner, 2017). The *z*-curve analysis also relies on assumptions about how the *p-*values (transformed into *z* values) distribute, that is, the fact that publication bias should give results characterized by an unusually large proportion of *p-*values that fall just below the 0.05 significance level (Bartoš and Schimmack, 2020). It also explicitly incorporates a random effect model (and thus can handle effect sizes heterogeneity) using a mixture of *z* distributions (Brunner and Schimmack, 2020). Furthermore, the *z*-curve provides two power estimates that allow a better estimate the replicability of the included studies (Bartoš and Schimmack, 2022): (1) the conditional average power of the studies yielding significant effects, called the expected replication rate (ERR), which is equivalent to the *p*-curve power estimate, and (2) the unconditional average power of the studies in the literature, called the expected discovery rate (EDR), which is the overall probability of obtaining significant effects when both significant and non-significant results are present in a literature. When this estimate is compared with the Observed Discovery Rate (ODR), that is, the proportion of statistically significant results within the z-curve analysis, an indicator of publication bias is obtained.

#### 2.5.3 Meta-analyses

The meta-analyses were conducted using the *RoBMA* package (Bartoš and Maier, 2020) in JASP and the *metafor* package in R using a restricted maximum-likelihood estimator method. They were based on the Hedge’s *g* effect size (and related SE and V) for the comparison of the TMS-dependent change in semantic control-related performance between the active and control TMS groups/conditions, as described above (see Data extraction).

In order to achieve maximum statistical power, we chose to use all the available effects of interest in the included studies, as noted above (see Data extraction). However, multiple effects extracted from the same study are expected to be more similar to each other than effects from different studies. Ignoring this effect size dependency tends to underestimate SE, which in turn results in an inflated type-I error rate (Hedges, 2009). Therefore, we performed a three-level random effects model using the *rma.mv* function, which models three sources of variance to account for effect size dependency, which was also performed with a cluster-robust variance estimation method using the *robust* function and the *clubSandwich* package (for more details, see Assink and Wibbelink, 2016; Assink and Wibbelink, 2023).

The random effects model allows evaluating the presence of publication bias with the rank test, but it does not provide tools to evaluate (and correct) the impact of the potential publication bias on the combined effect size resulting from the research synthesis. Therefore, we also employed other meta-analytical approaches (see below) that allowed us to do that, but without taking into account effect size dependency, after having performed a likelihood ratio test to verify whether the inclusion of the study grouping variable (to estimate the random variation between effect sizes from the same study and thus account for the effect sizes dependency) was justified.

First, a classical frequentist model was fitted using a random model, providing standard methods to evaluate the impact of publication bias (that is, the funnel plot with the trim-and-fill method in case of asymmetry, evaluated with the rank test and the Egger’s test). We then performed a frequentist meta-analysis using a two-sided selection model with one-tailed *p*-value cutoffs of 0.05 and 0.1 to evaluate the presence of heterogeneity and to evaluate and correct for the impact of selection bias around statistical significance (Vevea and Woods, 2005). This frequentist meta-analysis was complemented by a robust Bayesian meta-analysis calculated with the RoBMA package (Bartoš and Maier, 2020). Bayesian meta-analysis has the advantage of providing probabilities for the experimental and null hypotheses and additional tests for heterogeneity, as well as publication bias. As prior distributions, we used a normal distribution for the effect size (*μ* = 0, *σ* = 1), an inverse gamma distribution for heterogeneity (*α* = 1, *β* = 0.15), and the cumulative sum of the Dirichlet distribution (α = 1,1) for the two-interval selection model (with one-tailed p-value cutoffs of 0.05 and 0.1 for non-significant studies). Null priors were spike functions at 0. The study heterogeneity was then determined using standard measures (that is, the *Q* test and *τ*).

## 3 Results

### 3.1 Overview

The screening process sequence is depicted in the PRISMA flowchart (Figure 1). Initially, our literature search yielded a total of 85 records, of which 27 were unique records (i.e., after removing duplicates). Three additional articles were identified via citation searching. The resulting 30 full-text articles were retrieved and underwent full-text review. Ultimately, 16 studies met our inclusion criteria, involving a combined sample of 313 participants. These studies investigated a total of 35 effects (for a total sample of 688 participants), accounting for cases where the experimental design allowed to extract multiple effects (Davey et al., 2015; Häuser et al., 2016; Hoffman et al., 2012; Hoffman and Crutch, 2016; Medaglia et al., 2018, 2021; Timofeeva et al., 2024) or when multiple stimulation sites were used (Davey et al., 2015; Krieger-Redwood et al., 2014; Timofeeva et al., 2024; Wawrzyniak et al., 2017; Whitney et al., 2011, 2012; Zhang et al., 2019; Zhao et al., 2021). Note that a power analysis performed with the *metapower* R package revealed that our sample size (35 effects with a study size of *n* = 20) ensured a statistical power of about 80% to find an expected small/medium effect size of 0.35 with a random effect model, assuming a moderate/substantial heterogeneity (*I*^2^ = 0.60).

Response times served as the dependent variable across all studies, with 2 employing a between-participants design and 14 using a within-participants design. As regards the TMS stimulation protocols, the studies exhibited considerable homogeneity. Regarding the timing of stimulation, 3 studies targeted brain areas during task performance (i.e., online stimulation), while 13 targeted brain areas before the task (i.e., offline stimulation). The stimulation paradigms varied: of the 3 studies employing online TMS, 1 used triple-pulse TMS (40 Hz) (Zhang et al., 2019) and 2 used double-pulse TMS (25 Hz and 40 Hz, respectively, Teige et al., 2018, Zhao et al., 2021); of the 13 studies employing offline TMS, 9 used repetitive low-frequency TMS (1 Hz) (Häuser et al., 2016; Hoffman et al., 2010, 2012; Hoffman and Crutch, 2016; Krieger-Redwood and Jefferies, 2014; Davey et al., 2015; Whitney et al., 2011, 2012) and 4 used continuous theta burst TMS (3 50–Hz pulses at 5 Hz) (Medaglia et al., 2018, 2021; Timofeeva et al., 2024; Wawrzyniak et al., 2017). Brain regions of interest were localized on structural T1-weighted MRI scans for all participants. See Table 1 for more details.

**Table 1:** Characteristics of the studies included in this meta-analysis.

Study ID	DOI	Effect ID	Stimuli	Task	SCP type^a^	TMS protocol	Site	TMS intensity	TMS parameters	Design	Extracted effect	n
1	10.1523/JNEUROSCI.3783-10.2010	1	Words	Synonym judgment	c, d	rTMS, offline	l IFG	120% rMT (by eye)	1 Hz, 600 s	WTN, 3 × 2 × 2	TMS × SCP × TASK	13
2	10.1093/cercor/bhq180	2	Words	Association task	a	rTMS, offline	l IFG	120% aMT (by eye)	1 Hz, 600 s	WTN, 3 × 2 × 2 × 2	TMS × SITE × SCP × TASK	16
		3	Words	Association task	a	rTMS, offline	l pMTG	120% aMT (by eye)	1 Hz, 600 s	WTN, 3 × 2 × 2 × 2	TMS × SITE × SCP × TASK	16
3	10.1080/02687038.2011.608838	4	Pictures	Association task	b	rTMS, offline	l pMTG	120% rMT (by eye)	1 Hz, 600 s	WTN, 2 × 3 × 2	TMS × SITE × TASK	14
		5	Words	Association task	b	rTMS, offline	l pMTG	120% rMT (by eye)	1 Hz, 600 s	WTN, 2 × 3 × 2	TMS × SITE × TASK	14
4	10.1162/jocn_a_00123	6	Words	Association task	b	rTMS, offline	l IFG	120% aMT (by eye)	1 Hz, 600 s	WTN, 3 × 2 × 5	TMS × SITE × TASK	16
		7	Words	Association task	b	rTMS, offline	l pMTG	120% aMT (by eye)	1 Hz, 600 s	WTN, 3 × 2 × 5	TMS × SITE × TASK	16
		8	Words	Association task	b	rTMS, offline	l IPL	120% aMT (by eye)	1 Hz, 600 s	WTN, 3 × 2 × 5	TMS × SITE × TASK	16
5	10.1016/j.neuropsychologia.2014.09.014	9	Pictures	Cycling picture naming	b	rTMS, offline	l IFG	120% rMT (by eye)	1 Hz, 600 s	WTN, 2 × 2 × 2 × 6	TMS × SCP	16
		10	Pictures	Cycling picture naming	b	rTMS, offline	l pMTG	120% rMT (by eye)	1 Hz, 600 s	WTN, 2 × 2 × 2 × 6	TMS × SCP	16
6	10.1523/JNEUROSCI.4705-14.2015	11	Word-picture	Word-picture matching	a	rTMS, offline	l pMTG	120% aMT (by eye)	1 Hz, 600 s	WTN, 3 × 4 × 2	TMS × SCP	18
		12	Word-picture	Word-picture matching	a	rTMS, offline	l IPL	120% aMT (by eye)	1 Hz, 600 s	WTN, 3 × 4 × 2	TMS × SCP	18
		13	Word-picture	Identity-matching task	b	rTMS, offline	l pMTG	120% aMT (by eye)	1 Hz, 600 s	WTN, 3 × 4 × 2	TMS × SCP	18
		14	Word-picture	Identity-matching task	b	rTMS, offline	l IPL	120% aMT (by eye)	1 Hz, 600 s	WTN, 3 × 4 × 2	TMS × SCP	18
7	10.1016/j.neuropsychologia.2016.09.012	15	Words	Feature selection	b	rTMS, offline	l IFG	100% aMT (by eye)	1 Hz, 600 s	WTN, 3 × 2	SITE × SCP	18
8	10.1016/j.neuropsychologia.2016.09.003	16	Sentences	Meaningfulness judgment	a	rTMS, offline	l IFG	110% aMT (by eye)	1 Hz, 600 s	WTN, 2 × 2 × 2	SITE × SCP	16
		17	Sentences	Meaningfulness judgment	a	rTMS, offline	l IFG	110% aMT (by eye)	1 Hz, 600 s	WTN, 2 × 2 × 2	SITE × SCP	16
9	10.1016/j.cortex.2015.11.021	18	Words	Taxonomic judgment	b	rTMS, offline	r IPL	65% max output	1 Hz, 600 s	WTN, 3 × 2 × 2	TMS × SITE × SCP	18
		19	Words	Synonym judgment	b	rTMS, offline	r IPL	65% max output	1 Hz, 600 s	WTN, 3 × 2 × 2	TMS × SITE × TASK	18
10	10.1371/journal.pone.0177753	20	Sentences	Lexical decision	d	cTBS, offline	l IFG	80% aMT (MEP)	3 50-Hz pulses at 5 Hz	WTN, 3 × 4	TMS × SCP	19
		21	Sentences	Lexical decision	d	cTBS, offline	l pMTG	80% aMT (MEP)	3 50-Hz pulses at 5 Hz	WTN, 3 × 4	TMS × SCP	19
11	10.1523/JNEUROSCI.0092-17.2018	22	Words/sentences	Verb generation/sentence completion	a	cTBS, offline	l IFG	80% aMT (by eye)	3 50-Hz pulses at 5 Hz	BTN, 2 × 2 × 2	TMS × SITE × SCP	28
		23	Words/sentences	Verb generation/sentence completion	a	cTBS, offline	l IFG	80% aMT (by eye)	3 50-Hz pulses at 5 Hz	BTN, 2 × 2 × 2	TMS × SITE × SCP	28
12	10.1016/j.cortex.2018.03.024	24	Words	Association task	a	dbTMS, online	l pMTG	60% max output	25 Hz	WTN, 2 × 3 × 4	TMS × SCP	15
13	10.1002/hbm.24781	25	Words	Association task	b	tbTMS, online	l IFG	100% rMT (MEP)	40 Hz	WTN, 4 × 2	SITE × TASK	24
		26	Words	Association task	b	tbTMS, online	l pMTG	100% rMT (MEP)	40 Hz	WTN, 4 × 2	SITE × TASK	24
		27	Words	Association task	b	tbTMS, online	r IPL	100% rMT (MEP)	40 Hz	WTN, 4 × 2	SITE × TASK	24
14	10.1523/ENEURO.0382-20.2021	28	Sentences	Sentence completion	a	cTBS, offline	l IFG	80% aMT (by eye)	3 50-Hz pulses at 5 Hz	BTN, 2 × 2 × 2	TMS × SITE × SCP	41
		29	Words	Word generation	a	cTBS, offline	l IFG	80% aMT (by eye)	3 50-Hz pulses at 5 Hz	BTN, 2 × 2 × 2	TMS × SITE × SCP	41
15	10.1523/JNEUROSCI.1355-21.2021	30	Videos	Gender voice discrimination	c, d	dbTMS, online	l IFG	50% max output	40 Hz	WTN, 3 × 2 × 2 × 8	SITE × SCP	26
		31	Videos	Gender voice discrimination	c, d	dbTMS, online	l pMTG	50% max output	40 Hz	WTN, 3 × 2 × 2 × 8	SITE × SCP	26
16	10.1093/cercor/bhae188	32	Pictures	Cued picture naming	e	cTBS, offline	l pMTG	80% aMT (by eye)	3 50-Hz pulses at 5 Hz	WTN, 2 × 2 × 3	SITE × SCP	15
		33	Pictures	Cued picture naming	e	cTBS, offline	l IPL	80% aMT (by eye)	3 50-Hz pulses at 5 Hz	WTN, 2 × 2 × 3	SITE × SCP	15
		34	Pictures	Cued picture naming	e	cTBS, offline	l pMTG	80% aMT (by eye)	3 50-Hz pulses at 5 Hz	WTN, 2 × 2 × 3	SITE × SCP	14
		35	Pictures	Cued picture naming	e	cTBS, offline	l IPL	80% aMT (by eye)	3 50-Hz pulses at 5 Hz	WTN, 2 × 2 × 3	SITE × SCP	14

^a^ Type of manipulations of the semantic control process (SCP) requirements (see Section 2.2); TBS, theta-burst stimulation; dpTMS, double-pulse TMS; tpTMS, triple-pulse TMS; l and r, left and right hemisphere; rMT and aMT, resting and active motor threshold; WTN, within-ss design; BTW, between-ss design; n, size of the sample used in the reported.

The tasks meeting inclusion criteria involved manipulations related to semantic ambiguity (Hoffman et al., 2010), competitor interference (Krieger-Redwood and Jefferies, 2014; Davey et al., 2015; Hoffman et al., 2012; Hoffman and Crutch, 2016; Whitney et al., 2012; Zhang et al., 2019), association strength (Hallam et al., 2016; Teige et al., 2018; Whitney et al., 2011), semantic violations (Wawrzyniak et al., 2017; Zhao et al., 2021), meaning dominance (Davey et al., 2015; Häuser et al., 2016; Medaglia et al., 2018, 2021), and context switching (Timofeeva et al., 2024). These manipulations were applied to words (Davey et al., 2015; Hoffman et al., 2010; Hoffman and Crutch, 2016; Medaglia et al., 2018; Teige et al., 2018; Whitney et al., 2011, 2012; Zhang et al., 2019), sentences (Häuser et al., 2016; Medaglia et al., 2018, 2021; Wawrzyniak et al., 2017), pictures (Davey et al., 2015; Hoffman et al., 2012; Krieger-Redwood and Jefferies, 2014; Timofeeva et al., 2024), and videos (Zhao et al., 2021).

### 3.2 Risk of bias assessment

The risk of bias for the selected studies was assessed based on the effects obtained from the data analysis performed in this meta-analysis. Our analysis revealed that the risk of bias must be interpreted cautiously. Indeed, it is important here to reiterate that, if we had included the domain related to the randomization in the overall bias evaluation, most studies would have been rated as having a high risk of bias. This is because only three studies (Timofeeva et al., 2024; Zhang et al., 2019; Zhao et al., 2021) were deemed to have a “low risk” of bias in the randomization domain. These studies ensured that participants were unaware of the intervention assignments by using a control condition (e.g., the vertex of the head) that matched the physical sensations of the experimental intervention.

**Figure 2:**
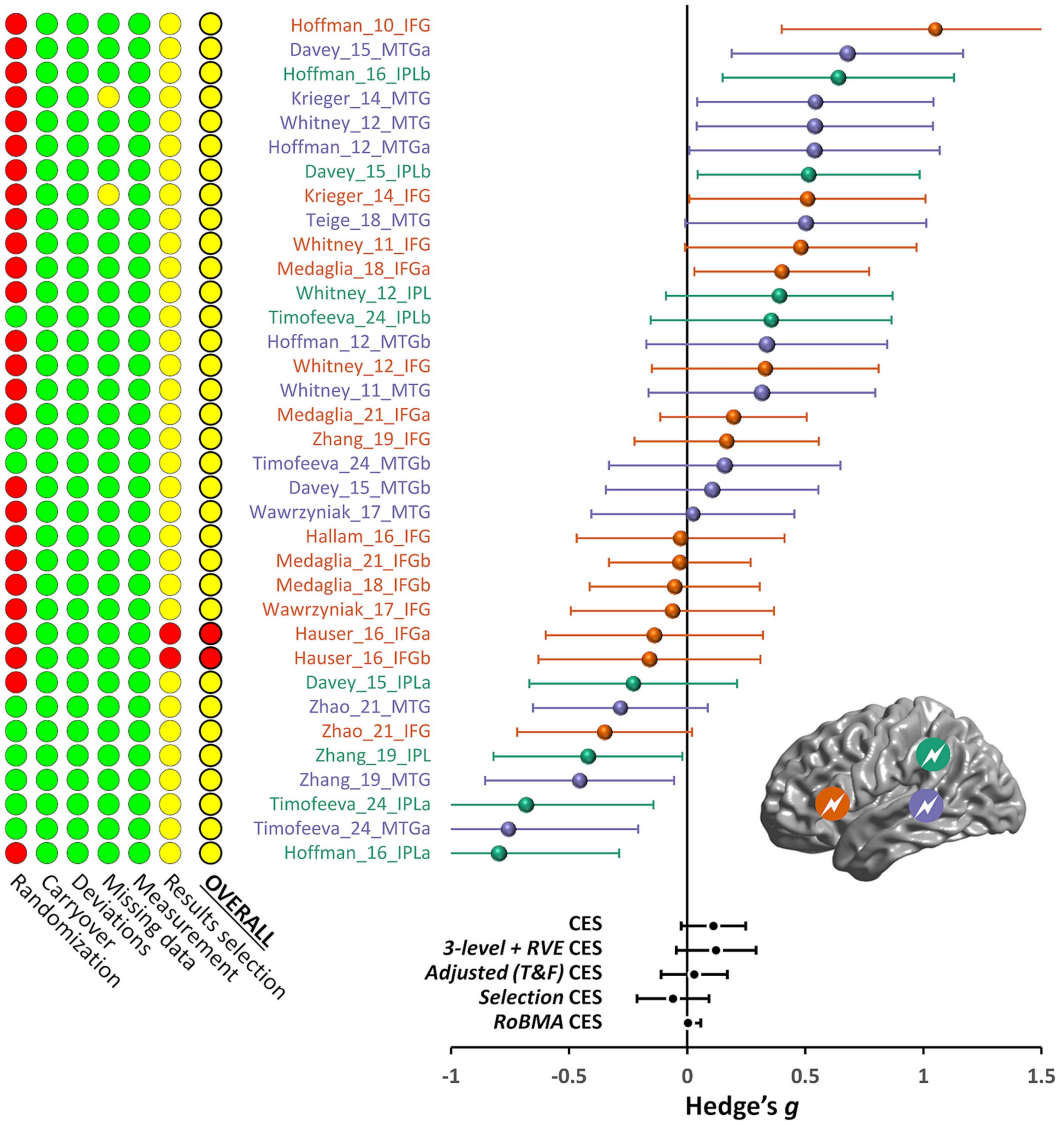
Risk of bias and effect sizes for the included effects. The traffic light plot on the left shows the risk of bias summary based on the authors’ judgments about each risk of bias item for each included study. Green, yellow, and red indicate low, unclear, and high risk of bias, respectively. See the main text for the description of the risk of bias dimensions. The forest plot on the right summarizes the meta-analysis results for all the included effects. Effects indicated in orange, purple, and green are derived from TMS stimulation of IFG, pMTG, and IPL, respectively (see inset); suffixes *a* and *b* indicate multiple effects from the same study and stimulated region. The bottom part of the forest plot shows the combined effect sizes (CES) derived from different meta-analytical approaches, as described in detail in the main text (*3-level + RVE*, three-level random effects model with a cluster-robust variance estimation method; *T&F*, trim-and-fill; *RoBMA*, robust Bayesian meta-analysis).

When the randomization domain was excluded from the overall evaluation, only one out of 16 studies (Häuser et al., 2016) had a “high risk” of bias due to concerns about selective reporting of results. The remaining 15 studies had “some concerns” in this domain because most authors had not prepublished their statistical analysis plan. The summary of the assessment performed in each of the six domains is given in Figure 2.

### 3.3 Results of synthesis and publication bias

**Figure 3:**
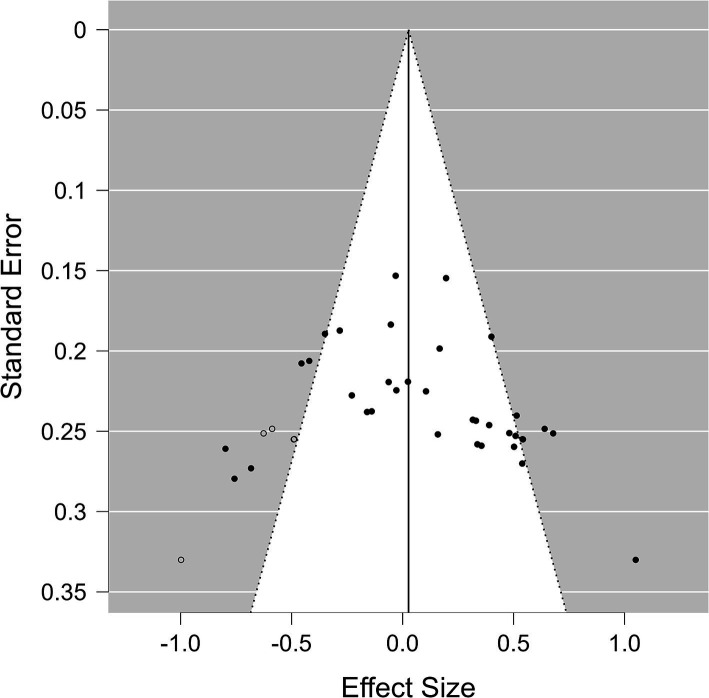
Trim-and-fill funnel plot. The funnel plot shows the effect sizes of the individual effects included in the meta-analysis as black dots. The empty dots represent the imputed and added effects after the trim-and-fill analysis.

We first present the results of the classical frequentist meta-analysis, which provides the standard methods to estimate the presence of publication bias. The effect sizes (and their standard error) for the comparison of the TMS-dependent change in semantic control performance between the active and control TMS groups/conditions are displayed in a funnel plot in Figure 3. Hedges’ g values for the included effects ranged from −0.80 to 1.05. The combined effect estimated by the random model was 0.111, with the CI_95%_ ranging from −0.026 to 0.248 (*Z* = 1.592, *p* = 0.111; see Figure 2). There appeared to be substantial heterogeneity among the true effects [*Q*(34) = 104.54, *p* < 0.001], suggesting that the effects of interest may differ widely across studies (*τ* = 0.341).

The three-level random effects models confirmed these results, with an estimated combined effect of 0.123, with the CI_95%_ ranging from −0.037 to 0.283 [*t*(34) = 1.567, *p* = 0.127; see Figure 2] and substantial heterogeneity among the true effects [*Q*(34) = 104.54, *p* < 0.001]. The results were essentially the same when using the cluster-robust variance estimation (*M* = 0.122, CI_95%_ = [−0.046–0.292], t(34) = 1.567, *p* = 0.140). However, it should be noted that the log-likelihood ratio test revealed that the inclusion of the random level for the studies, to account for effect size dependency, was not justified [𝛸(1) = 0.786, *p* = 0.375].

The funnel plot displayed in Figure 3 showed a slight asymmetry of the included effects (filled circles), as confirmed by the rank correlation test (Kendall’s τ = 0.267, *p* = 0.024) but not the Egger’s regression test (*Z* = 1.82, *p* = 0.069), suggesting that some publication bias might exist. The trim-and-fill method estimated that 5 studies were missing (empty circles). The combined effect size estimate obtained after their inclusion was thus even reduced (*M* = 0.03, CI_95%_ = [−0.11–0.17]; see Figure 2).

Regarding the heterogeneity selection models, since the test was significant, we proceeded with a random effect model (Bartoš et al., 2022). The point estimate of the combined effect was very close to that found in the classical meta-analysis, reported above (*M* = 0.111, CI_95%_ = [−0.024–0.246]). However, this analysis also revealed a significant publication bias [χ^2^(1) = 5.050, *p* = 0.025], indicating that non-significant results are less likely to be published compared to significant results. Adjusting for this publication bias led to a non-significant and negative effect size estimate (*M* = −0.060, CI_95%_ = [−0.213–0.092], z = −0.774, *p* = 0.439). Adjusted estimated heterogeneity was τ = 0.333 (the unadjusted one was τ = 0.228).

The results of the RoBMA analysis estimated the mean effect of the TMS-dependent change in semantic control performance between the active and control TMS groups/conditions and the corresponding CI_95%_ displayed in the forest plot in Figure 2. The model-averaged estimated combined effect size was g = 0.004 (median = 0), with a 95% credible interval of [0–0.057]. The analysis found strong evidence for the absence of the investigated effect (BF_01_ = 12.361) and strong evidence for the existence of publication bias (BF_10_ = 103.728). The best model was that including the publication bias but not the investigated effect and the heterogeneity (BF = 9.115).

**Figure 4:**
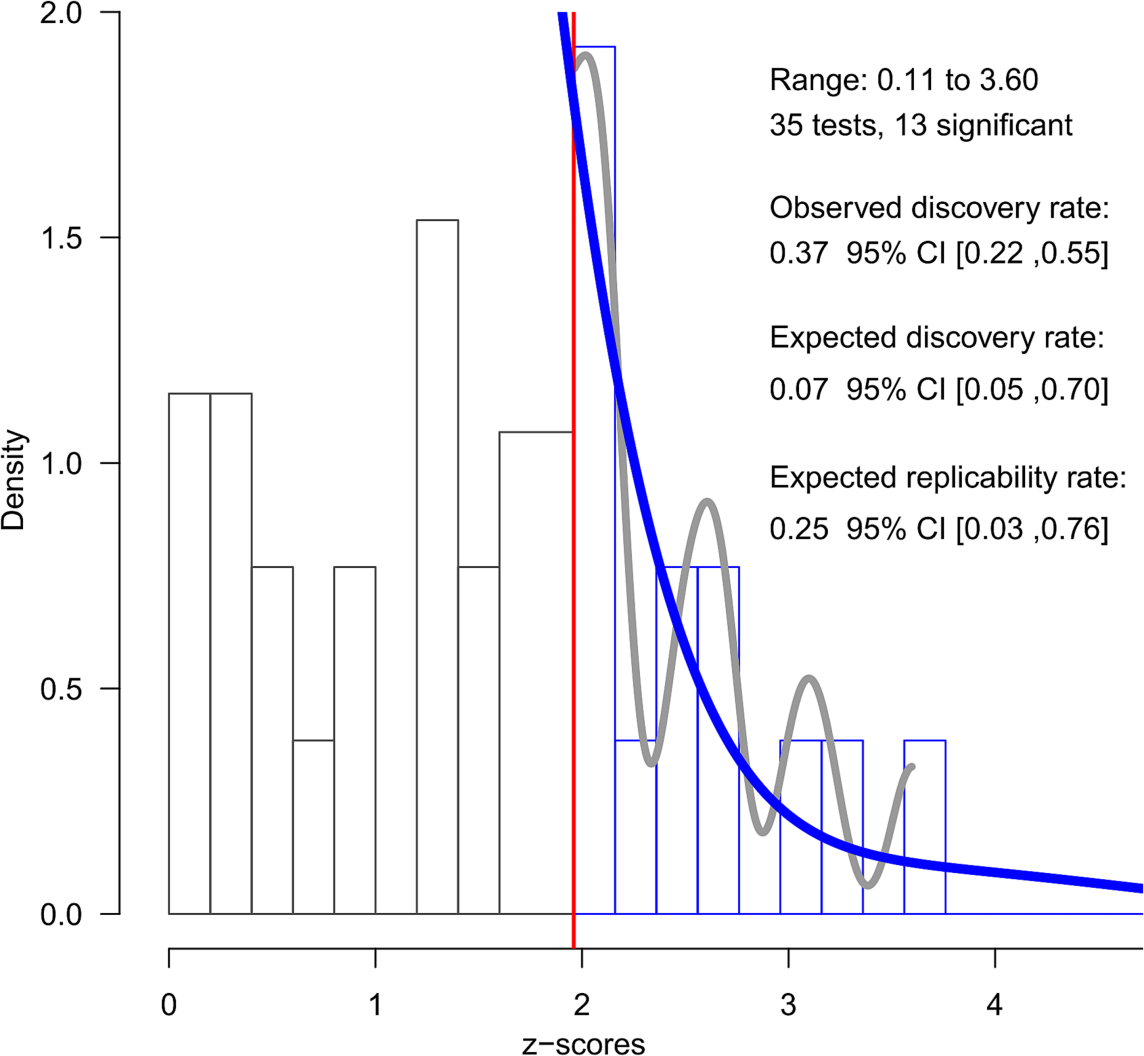
*Z*-curve analysis. The figure shows the results of the *Z*-curve analysis of the included effects converted into *z*-scores. The histogram displays the distribution of observed *z*-scores from the 35 effects included, with 13 being statistically significant (*z* > 1.96, indicated by the red vertical line). The grey line represents the observed density of the significant *z*-scores, while the solid blue line depicts the fitted *z*-curve model.

Regarding the z-curve analysis (see Figure 4), the conditional power of the significant results was estimated to be very low (ERR = 25%, CI_95%_ = [3–76%]); in other words, this analysis estimated that exact replication attempts of the included significant results would be expected to succeed 25% of the time. Furthermore, the unconditional power of any potential study was estimated to be even lower (EDR = 7%, CI_95%_ = [5–70%]), suggesting that only 7% of the studies would find a significant result. Since the observed discovery rate was considerably higher (ODR = 37%, CI_95%_ = [22–55%]) and its confidence interval did not include the EDR value, the results of this analysis provide statistically significant evidence for the existence of publication bias.

The sensitivity analysis confirmed the results reported above, showing that they were not dependent on our choice of the value for the correlation between repeated measures. Finally, we re-ran all the analyses reported above after excluding the effects related to IPL stimulation, because its inclusion in the multimodal semantic control network revealed by the Noonan and colleagues’ meta-analysis (2013) was not confirmed in a more recent meta-analysis (Jackson, 2021). The results reported above were substantially the same, confirming that there was no evidence to support a meaningful average effect and showing the presence of publication bias.

## 4 Discussion

This quantitative meta-analysis aims to assess the current state of research derived from transcranial magnetic stimulation (TMS) studies that allowed us to assess the predictions of the Controlled Semantic Cognition (CSC) framework (Lambon Ralph et al., 2017). Specifically, we examined evidence concerning the role of the inferior frontal gyrus (IFG), posterior middle temporal gyrus (pMTG), and inferior parietal lobule (IPL) in semantic control abilities (Lambon Ralph et al., 2017), as assessed by studies using inhibitory TMS interventions that are assumed to induce a reversible virtual lesion to these brain areas and, thus, to induce temporary semantic control deficits. To achieve this, we first identified all relevant studies published up until August 2024 in international journals (*N* = 16). We then assessed the robustness of their results using various analytical methods. Here, we discuss our primary findings and their implications in detail.

### 4.1 Robustness of evidence from TMS studies on semantic control abilities

The frequentist analysis revealed a small, non-significant combined effect size of TMS on semantic control processes. This result was accompanied by high variability (heterogeneity) across studies, which suggests that results differ substantially depending on specific experimental setups or conditions. Both these results do not support a consistent role of the three analyzed brain regions in semantic control. Given that many effects were derived from the same articles or laboratories, we performed a three-level random effects model accounting for effect size dependency. This confirmed the lack of significant effects across all three brain regions. These results were also confirmed by a selection model, and a robust Bayesian meta-analysis (RoBMA, Bartoš and Maier, 2020), a method that incorporates uncertainty and prior information, not only confirmed the absence of a significant effect but provided evidence for the absence of such an effect.

As regards publication bias, it was indicated by the slight funnel plot asymmetry in the classical random model, suggesting that approximately five studies with non-significant outcomes might be missing from the published literature. After applying the trim-and-fill procedure, which estimates and adjusts for missing studies, the average effect size was even reduced. We further scrutinized publication bias using a selection model and a *z*-curve analysis, which revealed a potential compromise in the evidential values of TMS effects on semantic control, indicating that significant findings may be overrepresented due to publication bias. Finally, the RoBMA analysis provided strong statistical evidence for the existence of publication bias, highlighting the need for caution in interpreting positive results.

These findings do not necessarily disprove the authors’ theoretical claims or suggest intentional misconduct. However, they highlight potential methodological issues, such as the adequacy of analysis and reporting. Therefore, readers should critically evaluate the reported successes. Still, these results seem to not support the contributions of the IPL, IFG and pMTG to semantic control processes as proposed by the CSC framework (Lambon Ralph et al., 2017) and evidenced by the Noonan and colleagues’ meta-analysis of fMRI findings (2013). This conclusion of a lack of involvement of these brain regions in semantic control processes is thus puzzling, especially for IFG and pMTG. Indeed, the contribution of these regions to semantic control has been confirmed in a number of neuroimaging studies and in a recent fMRI meta-analysis (Jackson, 2021), which, however, did not find an effect for IPL. This might be due to the fact that different parts of IPL have different roles in semantic cognition. For example, ventral angular gyrus is typically implicated in easier rather than harder tasks, suggesting a role in semantic representation rather than in semantic control. It seems that only the dorsal part of the angular gyrus and intraparietal sulcus has a domain-general control role (Fedorenko et al., 2013), a role that would probably fail to show up when participants’ performance in a semantic control task is compared with a general control task. It should also be noted that studies targeting IPL were fewer in number and they targeted IPL in both the hemispheres. This could have decreased the effect size for IPL since the effect is supposed to be stronger in the left hemisphere according to the CSC framework. Still, our findings were confirmed even after excluding IPL outcomes, suggesting that their inclusion did not bias our conclusions.

A more straightforward interpretation of our findings is that the inhibitory TMS stimulation applied in the included studies over these areas was not effective in impairing participants’ performance under high semantic control demands. Indeed, while our results show no significant effects of TMS, the methodological variability in the experimental designs across studies may have further reduced the likelihood of detecting consistent effects. Moreover, evidence of publication bias suggests that non-significant results may be underrepresented in the literature. These findings raise questions about the reliability of TMS in simulating deficits in semantic control and highlight the need for methodological improvements in future studies.

### 4.2 Methodological strengths and limitations of the TMS studies

Some key aspects of the methodological soundness and homogeneity of the selected TMS studies should be highlighted because they contribute to strengthening the conclusions of the meta-analysis. To begin with, it is imperative to acknowledge that most of these studies employed an offline inhibitory TMS (13 out of 16 studies), facilitating cross-study comparisons. Second, all studies employed individualized structural imaging guidance that increases the efficacy of locating stimulation sites over scalp-based targeting methods (Beynel et al., 2019; Sack et al., 2009). This method considers interindividual differences in brain anatomy and is more accurate for fine-grained targeting. Reliable identification of the sites is the first step in a successful understanding of the neural substrate underlying the process of interest. Another consistent and positive aspect in all analyzed studies was the amplitude dosing of the TMS stimulation based on the motor threshold of the participants (13 out of 16 studies). Although this may be inappropriate to guide amplitude stimulation in non-motor areas of the brain, it still considers individual differences in the physiological response induced by stimulation. Finally, to ensure that the observed results could not be explained by the nonspecific effects of the TMS procedure or the general difficulty of the task, eleven studies included a control task, such as a number judgment (Hoffman et al., 2010; Zhang et al., 2019), number naming (Medaglia et al., 2018, 2021) and Navon (Whitney et al., 2011, 2012) tasks. The use of a control task offers significant advantages by elucidating the precise role of a specific brain region. Indeed, this methodology proves to be more insightful, as it enables a direct comparison between the effects of TMS on the experimental task of interest and the control task. The expectation here was that TMS should manifest an impact on the target task, which involves the semantic control process, while leaving the control task relatively unaffected (e.g., the number judgment task).

However, we point out that most of these studies were short in power as they used fairly small sample sizes, as supported by the *z*-curve analysis. This limitation is exacerbated by the employment of the same participants under multiple conditions and experiments in some studies. These shortcomings could increase the risk of finding false negatives and inflated effect sizes (Button et al., 2013; Ioannidis, 2005), ultimately undermining result reliability and replicability.

The risk of bias in selecting the results reported in most TMS studies has also emerged. However, it should be noted that this factor raises ‘some concerns’ in a study even only if the data analysis plan is not pre-registered. The practice of pre-registration, which was notably infrequent during the era in which several of these studies were undertaken, has emerged in recent years as an increasingly esteemed methodology within the realm of research. This paradigm shift toward pre-registration can be attributed to its manifold advantages, foremost among them being the safeguard against the common pitfall of researchers tailoring their results to fit the data, thus mitigating the risk of overfitting. Additionally, it serves as a powerful instrument in augmenting the transparency and methodological rigor of research endeavors, thereby fortifying the foundations upon which scientific conclusions are built. Furthermore, pre-registration provides a unique opportunity to meticulously scrutinize *a priori* theories, affording scholars a means to assess hypotheses empirically and comprehensively before the onset of data collection, fostering a more robust scientific discourse.

Randomization and allocation concealment, critical to reducing bias, were generally not feasible in TMS studies., In TMS studies, participants and, especially, experimenters are likely aware of the type of stimulation being administered. Indeed, participants can often distinguish between real stimulation, which induces a stronger physical sensation at the stimulation site, and sham stimulation. Additionally, experimenters always know the condition (e.g., site and type of TMS stimulation) they are administering.

Another methodological issue in TMS studies is the inconsistent settings and adjustment for participants discomfort that may have reduced the comparability and efficacy of stimulation across studies. For example, in our meta-analysis, 13 out of 16 studies used stimulation intensities ranging from 80 to 120% of the active or resting individual motor threshold, while three studies used fixed stimulation intensities for all participants. In both cases, it remains unclear if these measures are the most reliable for stimulating areas outside the motor cortex. Furthermore, in some studies, the stimulation intensity was reduced due to participants experiencing pain sensations (Häuser et al., 2016; Whitney et al., 2011, 2012).

### 4.3 Methodological recommendations

Recent practices and recommendations for psychological studies could also be adopted in this specific field without compromising methodological rigor. As previously mentioned, most TMS studies on semantic control are severely underpowered due to relatively small sample sizes, with a few exceptions (Zhang et al., 2019; Medaglia et al., 2018, 2021). It is worth noting that Medaglia et al.’s studies used a between-subject design, which is known to be less powerful than a within-subject design. All the included studies had sample sizes smaller than 20, which is associated with low statistical power. This lower power increases the likelihood of false negatives and overestimation of true effect sizes (Button et al., 2013; Ioannidis, 2005). Larger sample sizes are crucial for obtaining reliable and valid results. Small samples are also more susceptible to the researcher’s degrees of freedom (e.g., trying several procedures of outlier exclusion and data analyses, etc.), which increases the probability of obtaining significant results by chance (Simmons et al., 2011).

A third way to potentially improve research on semantics methodologically, and consequently enhance our understanding of semantic control, is to preregister the study hypothesis, sample size, and analysis plan in repositories like Open Science Framework^2^ and Protocols.io^3^ before starting the experiment (for details, see Simmons et al., 2021). Researchers should also consider publishing their studies as registered reports (i.e., articles accepted before data collection and analysis, provided they meet required quality standards) (Chambers and Tzavella, 2022). This approach will facilitate the dissemination of negative and null results and prevent p-hacking and HARKing (Simmons et al., 2021) thereby reducing risks associated with publication bias.

Finally, we observed that most of the selected studies (e.g., Whitney et al., 2011, Krieger-Redwood and Jefferies, 2014; Hallam et al., 2016) used analyses of variance. However, in psycholinguistic and neurolinguistic research, participants are often presented with lists of linguistic stimuli, and researchers aim to draw general conclusions that extend beyond the specific sample and the set of items used. Linear mixed-effects modeling would be a more appropriate approach for analyzing this type of data, offering several advantages over traditional general linear model analyses (such as repeated measures analysis of variance and multiple regression). Unlike general linear models, mixed-effects models do not require prior averaging across participants and items, thus preserving and considering their variability (Montefinese et al., 2014; Visalli et al., 2023; Viviani et al., 2024). This approach increases the accuracy and generalizability of parameter estimates, allowing for a better evaluation of the effects of predictors (i.e., variables of interest and confounding factors, such as word frequency and length) and providing stronger protection against capitalization on chance, or Type I error (Baayen et al., 2008; Quené and van den Bergh, 2008). Therefore, a final recommendation for future neurostimulation studies on semantic control processes is to adopt linear mixed-effects models as a standard practice in their analysis routine, as it will enhance the credibility of their outcomes.

## 5 Conclusion

In this meta-analysis, we examined TMS studies targeting the IFG, pMTG, and IPL to assess their role in semantic control. Our results seem to challenge the contributions of IFG and pMTG to semantic control processes as proposed by the CSC framework (Lambon Ralph et al., 2017) and the fMRI meta-analysis by Noonan et al. (2013). This is puzzling, given the strong evidence from fMRI studies for these regions’ roles in semantic control. However, our findings may reflect limitations in TMS methodology rather than an actual absence of functional contributions by these regions. One plausible explanation for the lack of significant findings is that the inhibitory TMS protocols used in these studies may not have effectively disrupted participants’ performance on tasks requiring high semantic control. Methodological variability—such as differences in task design and stimulation protocols—might have limited the reliability of TMS in simulating deficits in these processes and raises concerns about the replicability of the observed effects. Furthermore, our study revealed stronger evidence for the existence of publication bias, raising questions about whether the literature represents the full scope of TMS outcomes. Future studies should adopt more rigorous methodologies, including larger sample sizes, pre-registration of study designs, and advanced statistical techniques to enhance the reliability of TMS as a tool for investigating the neural mechanisms underlying semantic control.

## Data Availability

The data analyzed in this study is subject to the following licenses/restrictions: the data for the meta-analysis have been derived from previously published studies. Requests to access these datasets should be directed to Maria Montefinese, maria.montefinese@unipd.it.

## Footnotes

^1^https://automeris.io/WebPlotDigitizer/

^2^https://www.cos.io/initiatives/prereg

^3^https://www.protocols.io/
